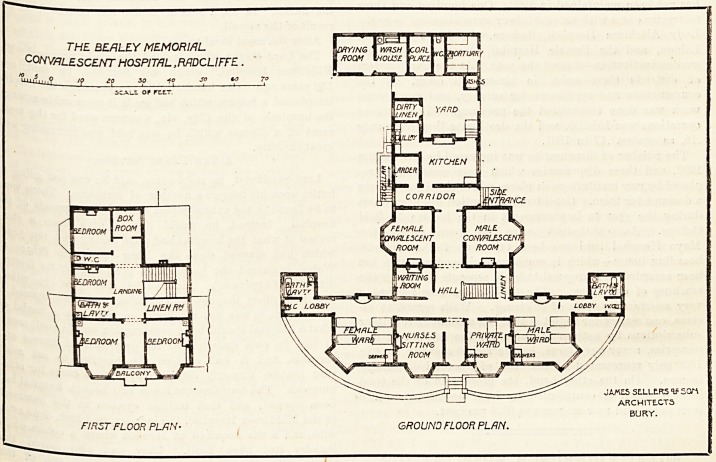# Convalescent Hospital, Radcliffe

**Published:** 1904-05-07

**Authors:** 


### HOSPITAL ADMINISTRATION.

### CONSTRUCTION AND ECONOMICS.

### CONVALESCENT HOSPITAL, RADCLIFFE.

FOR this hospital the town of Radcliffe is indebted to the munificence of Mr. Crompton Bealey, of the Manor House, Bury, whose family have lived there for at least five generations, and have been identified with one of the Lancashire industries during the whole of that period.

The ground plan of the hospital resembles the letter T, with the horizontal portion of the letter, facing the south. The main entrance occupies the centre of this part, and on the right hand side on entering are a private ward for one bed and the men's ward for four beds. From this ward projects a small block containing bath-room, lavatory, and closet; which are properly cut off by a cross-ventilated passage. On the left side of the entrance are the nurses' sitting-room, and a four-bedded ward for women, with similar bath-room accommodation to that for the men. These four-bedded wards apparently contain about one hundred superficial feet of floor space for each bed, and this is just about enough, and, assuming that the wards are 12 feet high, would give 1,200 cubic feet to each patient. To maintain the requisite standard of purity of air, it will be necessary to renew the air in the ward at least three times an hour. To ensure this there is a fair amount of cross-ventilation in the long axis of the ward; and, further, the elevation shows that Boyle's or other extractors exist.

Behind the sitting-room and private ward runs a corridor, and further back are the staircase and waiting-room, and beyond these are the men's and the women's convalescent rooms—these rooms occupying a somewhat unusual position exactly opposite each other. A second corridor runs at the back of these rooms, and here is the side entrance to the hospital. Beyond this corridor are the kitchen and laundry departments, and both these are well and compactly arranged.

FIRST FLOOR PLAN.

GROUND FLOOR PLAN.

The first floor contains four bedrooms, bath-rooms, linen-room, and box-room.

Speaking generally, it may be said that the plan is a fairly good one, and that it reflects some credit on its designers. The least satisfactory point is the conformation of the four-bedded dormitories. These are only about 18 feet wide, and with bedsteads 6 feet 6 inches long, would give only 5 feet between the ends of the beds, which is hardly enough. Then the shape of the wards necessitates that the amount of wall space for the two beds is not more than 11 feet, which, with bedsteads 3 feet wide, would give 5 feet between the beds; but this objection is lessened by the fact that the other sides of the beds have plenty of free space. Still where there was $1\frac{1}{2}$ acre of building land, it seems a pity that these wards were not a few feet wider and a few feet longer.

The architects are Messrs. Sellers and Son, of Bury; and the contractor was Mr. John Allen, of Radcliffe. The cost was about £23,000.

## Figures and Tables

**Figure f1:**